# Multi-Harmonic Nonlinear Ultrasonic Fusion with Deep Learning for Subtle Parameter Identification of Micro-Crack Groups

**DOI:** 10.3390/s25041152

**Published:** 2025-02-13

**Authors:** Qi Lin, Xiaoyang Bi, Xiangyan Ding, Bo Yang, Bingxi Liu, Xiao Yang, Jie Xue, Mingxi Deng, Ning Hu

**Affiliations:** 1 School of Mechanical Engineering, Hebei University of Technology, Tianjin 300401, China; 202231206037@stu.hebut.edu.cn (Q.L.); dingxiangyan@hebut.edu.cn (X.D.); boyang@hebut.edu.cn (B.Y.); yangx520@hebut.edu.cn (X.Y.); xuejie@hebut.edu.cn (J.X.); ninghu@hebut.edu.cn (N.H.); 2 State Key Laboratory of Reliability and Intelligence Electrical Equipment, Hebei University of Technology, Tianjin 300401, China; 3 Tianjin Fire Science and Technology Research Institute of Ministry of Emergency Management, Tianjin 300381, China; liubingxi@tfri.com.cn; 4 College of Aerospace Engineering, Chongqing University, Chongqing 400044, China; mxdeng@cqu.edu.cn

**Keywords:** convolutional neural network, information fusion, micro-crack groups identification, nondestructive testing, ultrasonic nonlinearity

## Abstract

Fatigue crack defects in metallic materials significantly reduce the remaining useful life (RUL) of parts. However, much of the existing research has focused on identifying single-millimeter-scale cracks using individual nonlinear ultrasonic responses. The identification of subtle parameters from complex ultrasonic responses of micro-crack groups remains a significant challenge in the field of nondestructive testing. We propose a novel multi-harmonic nonlinear response fusion identification method integrated with a deep learning (DL) model to identify the subtle parameters of micro-crack groups. First, we trained a one-dimensional convolutional neural network (1D CNN) with various time-domain signals obtained from finite element method (FEM) models and analyzed the sensitivity of different harmonic nonlinear responses to various subtle parameters of micro-crack groups. Then, high harmonics were fused to perform a decoupled identification of multiple subtle parameters. We enhanced the Dempster–Shafer (DS) evidence theory used in decision fusion by accounting for different sensitivities, achieving an identification accuracy of 93.73%. Building on this, we assigned sensor weights based on our proposed new conflict measurement method and further conducted decision fusion on the decision results from multiple ultrasonic sensors. Our proposed method achieves an identification accuracy of 95.68%.

## 1. Introduction

With the repeated application of loads, groups of fatigue micro-cracks are nucleated in localized areas of metallic materials, which are one of the most common and critical types of defects. As the service time of the equipment increases, these micro-crack groups evolve into macro-crack groups, resulting in a sharp decline in the part’s safety performance and increasing the risk of fracture failure [1,2,3,4]. Therefore, accurately monitoring the evolution processes of micro-crack groups is crucial for predicting the remaining useful life (RUL) of the metallic parts and preventing catastrophic accidents [5].

Existing research on nonlinear ultrasonic detection technology has primarily focused on multi-parameter identification of single cracks. Micro-crack groups have typically been characterized by macroscopic single parameters such as density or intensity rather than subtle parameters like quantity, size, orientation, or distribution. Decoupled detection technology for the subtle parameters of micro-crack groups is rarely addressed. However, the quantity and size of the micro-cracks within a damaged area directly determine the distance between micro-crack tips. When these distances diminish to a certain extent, minor cracks coalesce into major cracks [6]. Additionally, the size of the micro-cracks determines the stress intensity factor (SIF) at the micro-crack tips, thereby influencing the crack growth rates [7]. Therefore, the quantity and size of the micro-crack groups are the key subtle parameters determining the RUL. Timely acquisition of subtle parameter information of the micro-crack groups is more helpful for us to predict the propagation behaviors of micro-cracks and track the evolution processes of micro-crack groups. This paper aims to identify the quantity and size parameters of micro-crack groups. Compared to traditional nondestructive testing methods, such as computed tomography, eddy current testing, and infrared thermography, ultrasonic testing has been widely adopted due to its advantages of low equipment costs, portability, and broad applicability. Traditional linear ultrasonic detection technologies are sensitive only to a macro-crack, whereas nonlinear ultrasonic detection technologies can capture a micro-crack with a size smaller than half the ultrasonic wavelength [8]. In recent decades, many researchers have verified the feasibilities of nonlinear ultrasonic detection technologies for micro-cracks based on various evaluation indicators [9,10,11,12]. However, the interaction mechanisms between ultrasonic waves and micro-crack groups are complex, making it challenging to directly reconstruct the subtle parameters of micro-crack groups from ultrasonic responses. Fortunately, deep learning (DL) models have been applied in crack prediction and identification due to their excellent feature extraction capabilities. Rautela et al. [13] accurately identified the size and location of cracks in aerospace materials by using convolutional neural networks (CNNs) to extract signal features. Sbarufatti et al. [14] successfully predicted the position and size of cracks by inputting the damage index into an artificial neural network (ANN). Additionally, Pyle et al. [15] input imaging results of cracked pipes obtained using annular plane waves into a 2D CNN to extract features, accurately identifying the crack sizes. Xu et al. [16] utilized the Lamb transformer (LT) network to train spectrum maps derived from wave-mixing signals and achieved the localization and evaluation of micro-cracks. These findings suggest that DL models hold promise as an effective method for the quantitative identification of subtle parameters of micro-crack groups. In this paper, we propose a new method to train 1D CNNs by utilizing high harmonics, achieving high identification accuracy for the quantity and size parameters of micro-crack groups. The second harmonic demonstrates better performance compared to the third harmonic.

However, in practical engineering detection, it is unrealistic to pre-know any subtle parameters of micro-crack groups, necessitating the simultaneous identification of multiple subtle parameters. It is challenging to perfectly capture all information about micro-crack groups using a single nonlinear response. To tackle this issue, we break the traditional thought held by scholars of using a single nonlinear response to identify cracks and propose a concept of multi-harmonic nonlinear response detection. We innovatively combine independent nonlinear responses, effectively integrating valuable information captured by different nonlinear responses and comprehensively utilizing the specific identification capabilities of different nonlinear responses.

For each sensor, we fused two nonlinear responses at the data level, feature level, and decision level. The Dempster–Shafer (DS) evidence theory, a classical information fusion method [17], was applied in decision-level fusion. However, due to the different credibility degrees of different nonlinear responses, it is challenging to directly fuse them through the classical DS evidence theory. It is necessary to assign weights before decision-level fusion. Sun et al. [18] determined the weights of different data sources according to accuracy and improved the classical DS evidence theory. Xiao et al. [19] assigned weights based on the information volume and the credibility degree of each evidence. We assigned weights based on the independent performance of each nonlinear response and transferred these weights to each subtle parameter label, achieving an improvement in the classical Dempster combination rule. This method improved the identification accuracy of each sensor.

However, due to the uncertainty and limitation of single sensors, misjudgments are prone to occur. Therefore, multi-sensor data fusion technologies have been widely applied in various fields, such as image processing [20,21], remote sensing [22,23], fault diagnosis [24], and more. In this paper, we further applied decision-level fusion at the sensor level based on the decision-level fusion results of multi-harmonic nonlinear response. However, the high conflict between different sensors exposes the limitations of the classical DS evidence theory [25]. Therefore, many researchers have modified the evidence source models before fusion. Murphy et al. [26] utilized an arithmetic averaging method to modify original evidence. Deng et al. [27] measured the similarity between evidence and applied weighted corrections to the original evidence. We propose a novel conflict measurement method and assign sensor weights according to the conflicts, thereby obtaining weighted average evidence (WAE). We then used the classical Dempster combination rule to combine the WAE, further improving the identification accuracy of the subtle parameters of micro-crack groups. This confirms that our method can effectively handle sensor conflicts. This work paves the way for intelligent nondestructive testing of micro-crack groups.

## 2. Materials and Methods

### 2.1. Bilinear Stress–Strain Model

**Figure 1:**
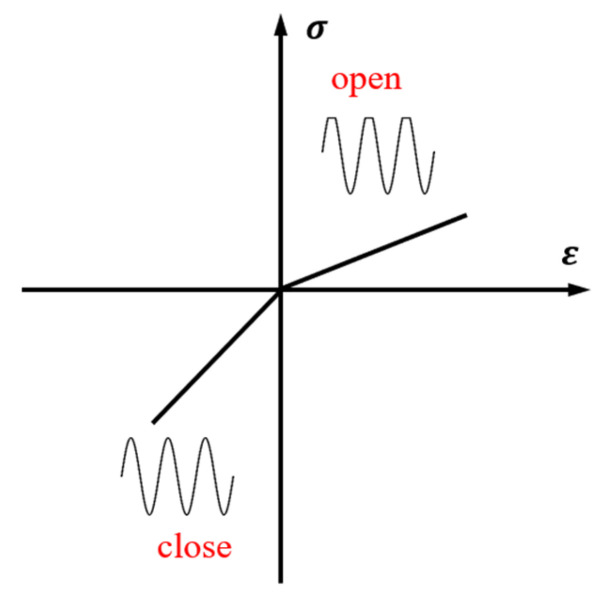
The bilinear constitutive relation between stress and strain.

Nonlinear ultrasonic techniques can detect the nonlinear effects induced by contact acoustic nonlinearity (CAN), hysteresis, and energy dissipation [28]. CAN primarily originates from the clapping effect or Hertzian contact [29]. When an ultrasonic wave encounters a micro-crack, the micro-crack closes under compression, increasing local stiffness, and opens under tension, decreasing local stiffness, as shown in Figure 1. The “clapping effect” of the micro-crack results in alternating changes in the local stress–strain relationship, which modulates the input ultrasonic wave and induces new harmonic components known as ultrasonic nonlinear responses [30,31].

### 2.2. Simulation Models

**Figure 2:**
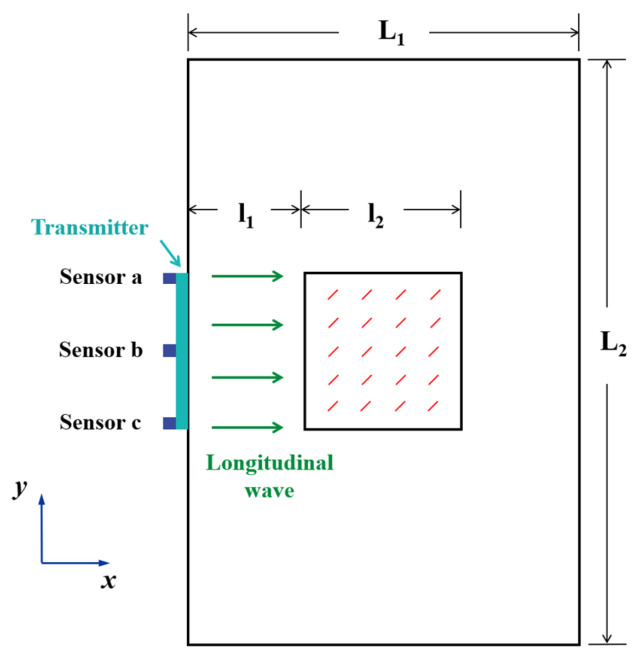
The FEM model.

Based on the bilinear stress–strain model, a numerical simulation method was employed in this paper. Two-dimensional finite element method (FEM) models containing micro-crack groups with different subtle parameters were established using Python (Version 2.7) and the commercial FEM software ABAQUS (Version 6.14). As shown in Figure 2, to simulate the micro-crack “clapping effect”, each crack in the FEM model was represented by two surfaces that could separate but not interpenetrate, employing a “hard contact” model in a normal direction. Considering the rough surfaces of micro-cracks in industrial equipment, the Coulomb law of friction (coefficient $\mu_{0}$ = 0.1) was applied in a tangential direction to simulate the friction behaviors of the micro-crack surfaces in this paper. The simulations utilized the material high-strength aluminum (AL-6061-T6) with a density of $\rho_{Al}$ = 2704 kg/m^3^ and Young’s modulus of $E_{Al}$ = 68.94 GPa. A square micro-crack group region with the side length $l_{2}$ = 40 mm was positioned at the model’s center. The left boundary of this region was set at a distance $l_{1}$ = 80 mm from the model’s left boundary. To mitigate the boundary reflection effects, the model’s length was set sufficiently long, resulting in the overall dimensions of $L_{1}\times L_{2}$ ($L_{1}$ = 200 mm and $L_{2}$ = 400 mm), as illustrated in Figure 2.

To excite the longitudinal plane waves modulated by a Hanning window, the dynamic displacement at the left end of the FEM model can be expressed as $u\left(x,t\right)=A_{0}\sin\left(2\pi f_{0}t/n\right)\cdot \sin\left(2\pi f_{0}t\right)$, where $A_{0}$ = 1 × 10^−3^ mm is the excitation signal amplitude, $f_{0}$ = 1 MHz is the center frequency, and n = 10 is the number of cycles per excitation signal. To balance computational efficiency and accuracy, the maximum element size was set to $L_{max}$ = 0.1 mm, not exceeding 1/20 of the excitation signal wavelength. Each micro-crack spanned at least 5 elements. The FEM model utilized four-node plane strain (CPE4R) elements. The ABAQUS/Explicit solver, based on the central difference method, simulated longitudinal plane wave propagation. Considering the need for numerical convergence and the total calculation time, the total analysis time was set to be 6.5 × 10^−5^ s, and the stable time increment Δ*t* was set to be 2 × 10^−9^ s. Three receiving points, serving as ultrasonic sensors a, b, and c were positioned at 20 mm intervals along the model’s left end to receive the original ultrasonic responses $U_{a}$, $U_{b}$, and $U_{c}$. The orientation angle of each micro-crack between the principal direction of the micro-crack and the positive direction of the x-axis was 45°. The spacing between the crack centers was maintained to be approximately equal, as shown in Figure 2.

### 2.3. Signal Preprocessing

**Figure 3:**
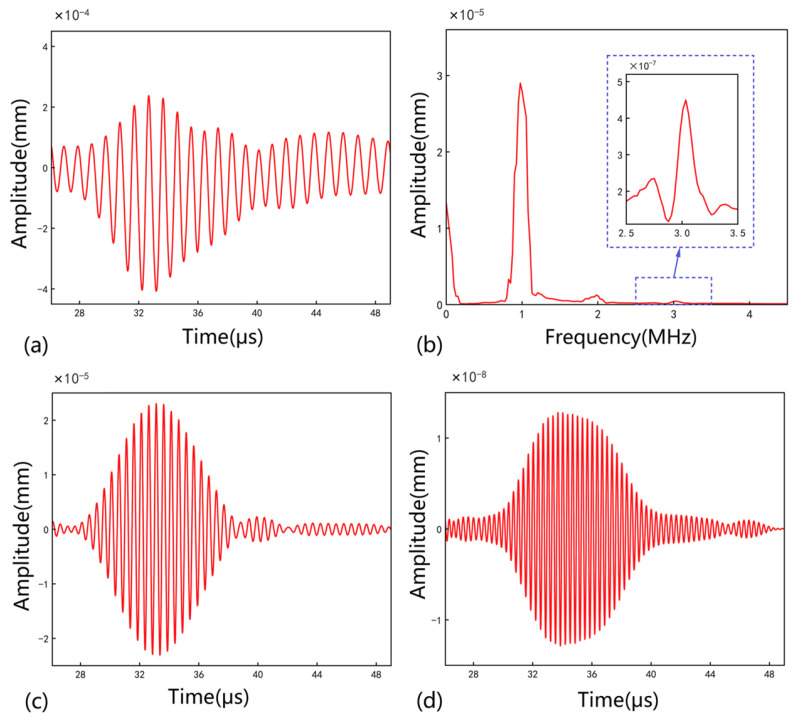
(**a**) The truncated $U_{b}’$. (**b**) The frequency domain. (**c**) The second harmonic. (**d**) The third harmonic.

To improve signal utilization and subsequent analysis efficiency, we optimized the simulation signals. The most significant time interval for the original ultrasonic responses $U_{a}$, $U_{b}$, and $U_{c}$ was calculated using Equation (1):

(1) $$\left[t_{1},t_{2}\right]=[\frac{l_{1}}{c_{1}}+\frac{l_{1}}{c_{2}}\;,\;\frac{l_{1}+l_{2}}{c_{1}}+\frac{l_{1}+l_{2}}{c_{2}}+t_{0}]$$

where $c_{1}$ ≈ 6127 m/s is the propagation velocity of the excited longitudinal plane waves, and $c_{2}$ ≈ 6127 m/s is the propagation speed of the crack-information-bearing response. This interval was determined to be [26.1 μs, 49.1 μs]. The original ultrasonic response signals were thus truncated to this interval, reducing the signal length from 15,000 to 4760 points, resulting in new ultrasonic responses $U_{a}’$, $U_{b}’$, and $U_{c}’$. Taking the FEM model with 210 micro-cracks of 0.8 mm as an example, the truncated $U_{b}’$ is shown in Figure 3a. The corresponding frequency domain is shown in Figure 3b. The second-harmonic nonlinear response was extracted from $U_{b}’$ using a Chebyshev bandpass filter, with the center frequency and the bandwidth 2 MHz and 0.2 MHz, respectively, as shown in Figure 3c. The third-harmonic nonlinear response corresponds to 3 MHz and 0.2 MHz, as shown in Figure 3d.

### 2.4. Labels and Sample Set Establishment

Prior to importing nonlinear responses into deep learning models, it is necessary to reasonably set subtle parameter labels and establish the sample sets. Considering the time and difficulty of sample collection, we set the minimum detectable size of the micro-cracks to be 0.22 mm. Additionally, to highlight the advantages of the nonlinear ultrasonic methods in detecting micro-cracks below half-wavelength, the maximum detectable size was set to 1.00 mm. The length of the subtle parameter interval determines the precision of identification (rather than accuracy. To balance identification precision and sample acquisition difficulty, we set the quantity parameter identification range between 10 and 300, divided into three contiguous intervals labeled “A”, “B”, and “C”. The size parameter identification range was divided into four contiguous intervals labeled “D”, “E”, “F”, and “G” (Table 1). Our goal is to classify more test samples into correct intervals to evaluate the RUL interval in conjunction with the life prediction model [32,33].

**Figure 4:**
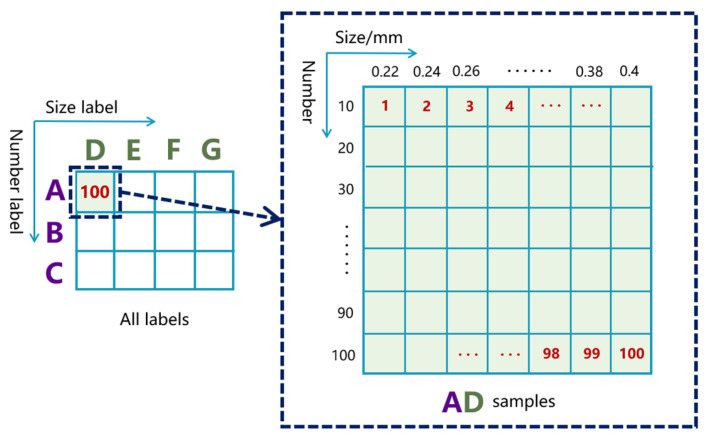
The overall subtle parameters space of the micro-crack groups.

Figure 4 illustrates the overall micro-crack group subtle parameters space. The space consists of 12 composite labels and 1200 samples. For example, the composite label “AD” (quantity label “A” and size label “D”) represents micro-crack quantities from 10 to 100 (step: 10) and sizes from 0.22 mm to 0.40 mm (step: 0.02 mm). This resulted in the generation of 100 different quantity-size combinations. Before parameter identification, in order to help readers understand how the amplitudes of the second and third harmonics change with differing quantities and sizes of micro-cracks, we calculated the integral values of the fundamental frequency component, second-harmonic component, and third-harmonic component in the frequency domain, denoted as $A_{1}$, $A_{2}$, and $A_{3}$, respectively. We used $A_{2}/A_{1}²$ and $A_{3}/A_{1}²$ to represent the Acoustic Nonlinearity Parameters (ANPs) for the second and third harmonics [34]. Table 2 and Table 3 show the relationships between these two ANPs (average of 100 samples) and the parameter labels. Clearly, under a different number of conditions, both ANPs increase with the size of the micro-cracks. The ANPs also change with variations in the quantity of micro-cracks, but no significant increasing or decreasing trend was observed.

When identifying a single subtle parameter, the overall FEM model set was split into four subsets based on the four size parameter labels, which were used as the FEM model sets to identify labels “A”, “B”, and “C” under the condition of different size parameters. Similarly, the overall FEM model set was split into three subsets based on the three parameter labels, which were used as the FEM model sets to identify labels “D”, “E”, “F”, and “G” under the condition of different quantity parameters. When identifying multiple subtle parameters, the overall FEM model set was used to identify 12 composite labels. All FEM models were divided into 90% for the training FEM models and 10% for the testing FEM models.

## 3. Results and Discussions

### 3.1. Single Subtle Parameter Identification

**Figure 5:**
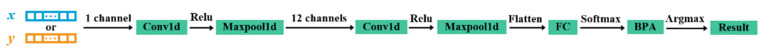
The identification framework for a single subtle parameter.

In order to explore the feasibility of deep learning methods for identifying micro-crack groups and lay the groundwork for the next work, we first conducted a test using single parameter identification. For single identification of the quantity parameter or the size parameter, we used one-dimensional convolutional neural networks (1D CNNs), which have achieved significant achievements in feature extraction and recognition of one-dimensional time series signals [35,36]. We utilized single harmonic nonlinear responses extracted from the FEM model set as the sample set and established a 1D CNN model for identifying single subtle parameters of micro-crack groups. Each sample, with a shape of (1, 4760), was input into the network through a single channel. As illustrated in Figure 5, the input sample was either x or y, depending on whether the second harmonic or the third harmonic from the FEM model set was selected.

For brevity, in this paper, the annotations following all convolutional layers indicate four hyperparameters: the number of input channels, the number of output channels, the convolutional kernel sizes, and the strides, respectively. For the max-pooling layers, they indicate the pooling kernel sizes and the strides. To enhance the neural network’s nonlinear capabilities, we applied a Rectified Linear Unit (ReLU) activation function after each convolutional layer. As shown in Figure 5, after initial optimization, the hyperparameters of each layer in the 1D CNN architecture along the direction of the arrows were set to Conv1d (1, 12, 3, 1), Maxpool1d (4, 2), Conv1d (12, 24, 3, 1), and Maxpool1d (4, 2).

The flattened output of the final max-pooling layer was fed into a fully connected layer (FC). The FC input shape is 7104, with the output shape varying based on the specific identification task (three neurons for quantity parameter identification and four neurons for size parameter identification). Then, the FC output was processed by a softmax layer to obtain probability distributions summing to 1. Finally, an argmax function then assigned the label with the highest probability as the identification label for each testing sample, which was compared with the true label to calculate the identification accuracy. What we have to be aware of is that the normal distribution standardization was used in the weight initialization, including all convolutional kernel weights and FC neuron weights. Additionally, all the initial biases were set to 1.

A mean square error (MSE) loss function and an Adam optimizer were used in the training process. To reduce 1D CNN’s iteration time and overfitting risks, the learning rate gradually decreased from 0.003 to 0.0001 over 20 steps. All neural networks were implemented using PyTorch (Python 2.7) on an GeForce RTX 4060 Laptop GPU (NVIDIA, Santa Clara, CA, USA).

**Figure 6:**
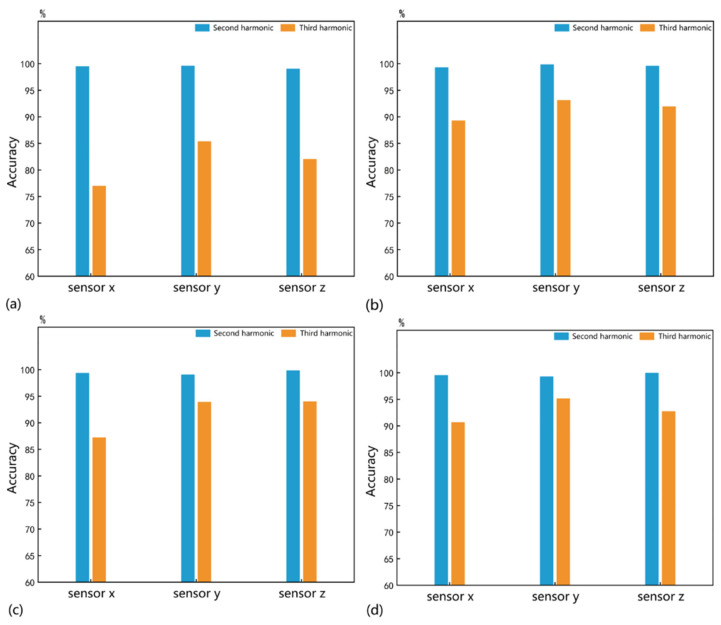
The mean identification accuracies of quantity parameters under the different size conditions of (**a**) 0.22–0.40 mm, (**b**) 0.42–0.60 mm, (**c**) 0.62–0.80 mm, and (**d**) 0.82–1.00 mm.

It took 6.14 s to train the 1D CNN for 600 epochs to identify the quantity parameter. The identification accuracy that stabilized with increasing iterations was recorded. To minimize errors, we randomly re-split the sample set and updated the initial weights of the neural networks, repeating this process 100 times. The mean identification accuracy is shown in Figure 6. Under all conditions, the identification accuracies for the quantity parameter by the second harmonic are greater than 99%, demonstrating a higher sensitivity than the third harmonic. Therefore, the 1D CNN driven by the second harmonic can be considered an effective method for identifying the quantity parameter of micro-crack groups.

**Figure 7:**
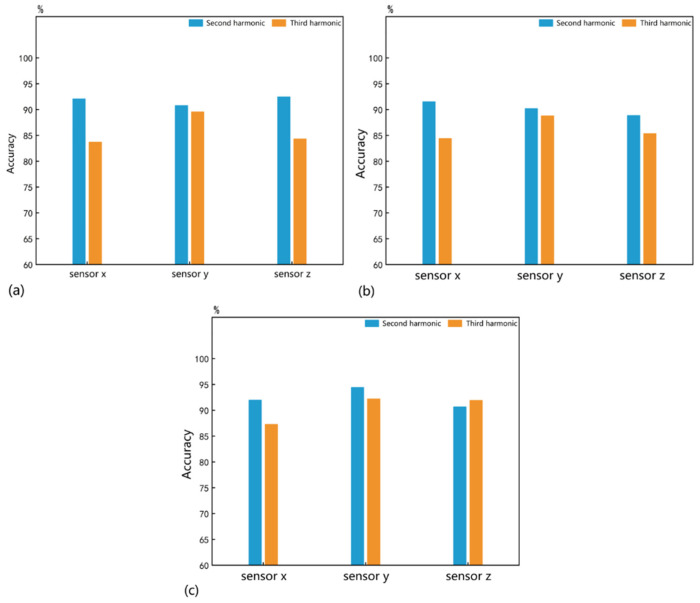
The mean identification accuracies of size parameters under the different quantity conditions of (**a**) 10–100, (**b**) 110–200, and (**c**) 210–300.

It took 8.16 s to train the 1D CNN for 600 epochs to identify the size parameter. As shown in Figure 7, the second harmonic exhibits higher sensitivity to the size parameter across all conditions. The highest identification accuracy of 94.42% was achieved by the second harmonic received by sensor b when the number of micro-cracks ranged from 210 to 300. However, size parameter identification performance is generally inferior to quantity parameter identification for all nonlinear responses.

The results of the test confirm that 1D CNN has a certain ability to identify subtle parameters of micro-crack groups and also demonstrate the significant advantages of the second harmonic in identifying the quantity and size parameters. However, when facing the problem of multi-parameter decoupling identification, it is uncertain whether the second harmonic can maintain a high identification accuracy. Therefore, the focus of our next work will be on dual-parameter decoupling identification.

### 3.2. Multiple Subtle Parameters Decoupled Identification

**Figure 8:**
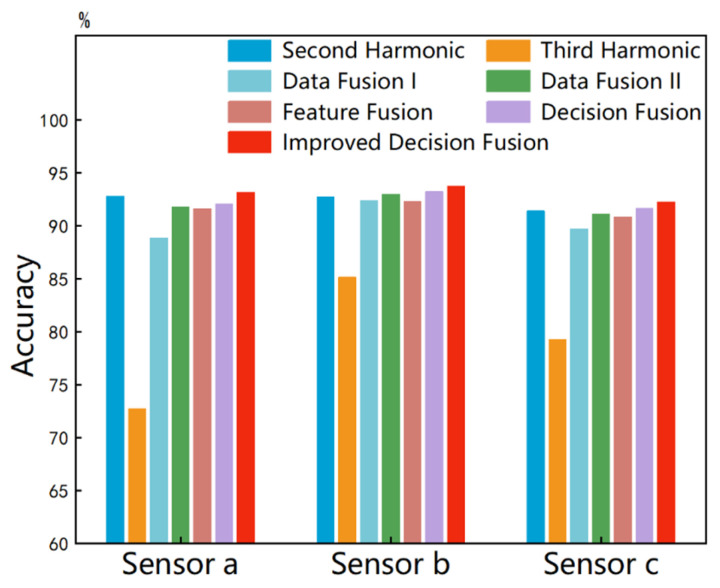
The mean decoupled identification accuracies for quantity and size parameters.

For the decoupled identification of the quantity and size parameters, we adapted the previous 1D CNN. The difference from the single subtle parameter identification is that the sample set has 12 labels. Consequently, the number of FC output layer neurons was adjusted to 12. It took 22.44 s to train the 1D CNN for 600 epochs. After repeating the training 100 times, the mean identification accuracies obtained are shown in Figure 8. The decoupled identification accuracies for the quantity-size parameters by the second harmonic under sensors a, b, and c are 92.69%, 92.78%, and 91.38%, respectively. However, the third harmonic only provides identification accuracies of 85.12%, 72.69%, and 79.26%. This indicates that although the second harmonic retains relatively high sensitivity, the addition of the second parameter undoubtedly reduces the identification accuracy of any nonlinear response. In addition, we supplemented the performance of the ultrasonic responses without bandpass filtering. The decoupled identification accuracies for the quantity-size parameters by the $U_{a}’$, $U_{b}’$ and $U_{c}’$ under sensors a, b, and c are 91.98%, 90.78%, and 91.28%, which confirms the necessity of extracting nonlinear responses through bandpass filtering.

### 3.3. Multi-Harmonic Fusion

To enhance the decoupled identification accuracy of multiple subtle parameters, we considered the specific mapping relationships between micro-crack groups and different nonlinear responses. Relying on a single nonlinear response is insufficient to comprehensively observe the subtle parameter information of micro-crack groups. Therefore, we innovatively combined independent nonlinear responses. Information fusion can effectively utilize multiple information sources to accurately identify targets. Based on this, we propose a series of new frameworks for the decoupled identification of multiple subtle parameters. For sensors a, b, and *c*, the second and third harmonics were fused at the data level, feature level, and decision level, respectively.

**Figure 9:**
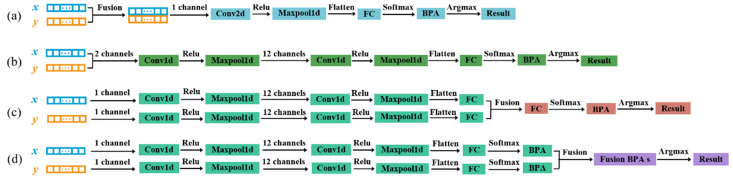
The decoupled identification framework at three levels for multiple subtle parameters: (**a**) Data-level fusionⅠ (data concatenating); (**b**) data-level fusionⅡ (multi-channel inputting); (**c**) feature-level fusion; (**d**) decision-level fusion.

Data-level fusion can make full use of raw data and overcome information loss [37,38]. As shown in Figure 9a, we propose a novel method of concatenating the second and third harmonics received by a single sensor. The fused second–third harmonic served as the sample set, where each sample has a shape of (2, 4760). Therefore, we established a 2D CNN and input the new samples through a single channel. The hyperparameters were set to Conv2d (1, 36, 2, 1), Maxpool2d (6), and FC (4758, 12). The annotations following all FCs indicate two hyperparameters: the input shape and the output shape. Valid padding was used in each convolutional layer to control the filter movement. In addition, other hyperparameters of the 2D CNN are similar to the previous 1D CNN. It took 32.94 s to train the 2D CNN for 600 epochs. The decoupled identification accuracies obtained over 100 training sessions, using the same method as before, are shown in Figure 8, with sensors a, b, and c achieving 92.32%, 88.84%, and 89.68%, respectively.

We also propose another data-level fusion method, as shown in Figure 9b. In this method, the second and third harmonics were fed into the 1D CNN through two separate channels. The hyperparameters were set to Conv1d (2, 12, 3, 1), Maxpool1d (4), Conv1d (12, 24, 3, 1), Maxpool1d (4), and FC (7104, 12). It took 24.92 s to train the 1D CNN for 600 epochs. The decoupled identification accuracies obtained over 100 training sessions, using the same method as before, are shown in Figure 8, with sensors a, b, and c achieving 92.96%, 91.74%, and 91.08%, respectively.

Different from directly fusing the raw data, feature-level fusion perceives and makes decisions by fusing deep features extracted from the raw data [39]. As shown in Figure 9c, the second and third harmonics were separately fed into two independent and identical 1D CNNs for feature extraction. The 1D CNNs used here are similar to the one shown in Figure 5, with the network architecture hyperparameters set to Conv1d (1, 12, 3, 1), Maxpool1d (4, 2), Conv1d (12, 24, 3, 1), Maxpool1d (4, 2), and FC (7104, 12). The outputs of the two FCs were algebraically summed to fuse features from different nonlinear responses. It took 43.32 s to train the two 1D CNNs for 600 epochs. The decoupled identification accuracies were obtained over 100 training sessions using the same method as before and are shown in Figure 8, with sensors a, b, and c achieving 92.31%, 91.58%, and 90.82%, respectively. Thus, it can be observed that neither data-level nor feature-level fusion has demonstrated a significant advantage. The specific reasons for this are not explored in depth in this paper.

Decision-level fusion is a high-level fusion method that synthesizes the independent judgment results from various information sources [40]. As shown in Figure 9d, the second and third harmonics were separately fed into two independent and identical 1D CNNs. The hyperparameters of each 1D CNN architecture were still set to Conv1d (1, 12, 3, 1), Maxpool1d (4, 2), Conv1d (12, 24, 3, 1), Maxpool1d (4, 2), and FC (7104, 12). The outputs of FCs were converted into basic probability assignments (BPAs) using two identical softmax layers. Next, we needed to fully utilize these BPAs. The DS evidence theory has significant advantages in dealing with uncertain and imprecise information [41]. We propose a decision fusion method for multi-harmonic nonlinear ultrasonic responses by utilizing DS evidence theory to fuse the BPAs provided by the second and third harmonics. For convenience, we denoted the second harmonic and the third harmonic as nonlinear response x and response y, respectively. We established a frame of discernment for storing various subtle parameter labels of micro-crack groups. From the previous part, it is easily understood that the quantity and size of the micro-cracks in any FEM model established in this paper are uniquely determined. Therefore, each sample corresponds to a unique determined label. We denoted the 12 mutually exclusive subtle parameter labels in Figure 4 as $\theta_{1},\theta_{2},\theta_{3},\ldots ,\theta_{12}$, representing AD, AE, …, CG, respectively. They formed the frame of discernment $\Theta^{s}$ under sensor s that is shown in (2). $P(\Theta^{s})$ is the power set consisting of elements from $\Theta^{s}$. The function m was defined as a mapping $m:P\left(\Theta^{s}\right)$ → [0, 1]. $m_{r}^{s}\left(\theta \right)$ represents the BPA value provided by the nonlinear response r under sensor s for θ, satisfying (3) (r=x,y;s=a,b,c). A high BPA value indicates a high degree of support.

(2) $$\Theta^{s}=\left\{\theta_{1},\;\theta_{2},\;\theta_{3},\ldots ,\;\theta_{12}\right\}$$

(3) $$\left\{\begin{array}{c} m\left(\Phi \right)=0\\ \sum_{\theta \subset P\left(\Theta^{s}\right)}m_{r}^{s}\left(\theta \right)=1 \end{array}\right.$$

(4) $$m^{s}\left(\theta \right)=\frac{1}{1-K^{s}}\sum_{\theta_{i}⋂\theta_{j}=\theta }m_{x}^{s}\left(\theta_{i}\right)m_{y}^{s}(\theta_{j})$$

(5) $$1-K^{s}=\sum_{\theta_{i}⋂\theta_{j}\neq \emptyset }m_{x}^{s}\left(\theta_{i}\right)m_{y}^{s}(\theta_{j})\;$$

We first used the classic Dempster combination rule, as shown in (4), where $K^{s}$ is the conflict coefficient between the second and third harmonics under the sensor s, as shown in (5). The BPA value $m^{s}\left(\theta \right)$ provided by sensor s for label θ is jointly determined by the second and third harmonics. The label with the highest BPA value was taken as the identification label. It took 33.02 s to train the two 1D CNNs for 600 epochs. As shown in Figure 8, the mean identification accuracies over 100 training sessions for sensors a, b, and c are 93.22%, 92.06%, and 91.65%, respectively. Surprisingly, compared to the simple recognition framework driven by the second harmonic alone, the decision fusion results based on the classic DS evidence theory do not perform well on any sensor.

### 3.4. Improvement of Multi-Harmonic Fusion

To find out the cause, we analyzed the fusion process of numerous testing samples. We found that the decision framework indiscriminately fused all the information provided by the two nonlinear responses. However, in the previous part, the identification performance of the third harmonic is inferior to that of the second harmonic. This means that the third harmonic, which carries more unreliable information, was given an equal opportunity to participate in the fusion process, severely misleading the decision-making process. To better understand this phenomenon, we illustrated the fusion process by taking a testing sample $S_{1}$ as an example, whose true label is $\theta_{1}$. The labels $\theta_{1}$, $\theta_{2}$, and $\theta_{3}$ satisfy:

$$\begin{array}{cc} m_{x}^{a}\left(\theta \right) & =\left\{m_{x}^{a}\left(\theta_{1}\right),\;m_{x}^{a}\left(\theta_{2}\right),\;m_{x}^{a}\left(\theta_{3}\right)\right\}\;=\;\{0.954,\;0.0452,\;9.16\times 10^{-4}\}.\\ m_{y}^{a}\left(\theta \right) & =\left\{m_{y}^{a}\left(\theta_{1}\right),\;m_{y}^{a}\left(\theta_{2}\right),\;m_{y}^{a}\left(\theta_{3}\right)\right\}\;=\;\{9.48\times 10^{-6},\;0.908,\;7.60\times 10^{-8}\}. \end{array}$$

What we have to be aware of is that we ignored the eight labels from $\theta_{4}$ to $\theta_{12}$ with extremely low BPA values, and approximately considered the sum of the BPA values of $\theta_{1}$ to $\theta_{3}$ as 1. Based on the classic DS evidence theory shown in (4) and (5), the fusion result is:

$$m^{a}\left(\theta \right)\approx \;\{2.213\times 10^{-4},\;0.9998,1.703\times 10^{-9}\}.$$

This adopts the suggestion of the third harmonic, leading to the misclassification of the test sample. Therefore, it is necessary to assign different weights to the two nonlinear responses. Under the condition of having previous experience, the independent performances of information sources under the same condition are usually used as a reference standard [18].

Based on the independent performances of the two nonlinear responses on the same 1D CNN from the previous part, as shown in (6), we denoted the mean identification accuracy of the nonlinear response r under sensor as $\mu_{r}^{s}$, which determines the credibility $\beta_{r}^{s}$ of this nonlinear response. Clearly, a nonlinear response with a higher mean identification accuracy should have a higher credibility and can be assigned a higher weight $w_{r}^{s}$, as shown in (7) (r=x,y;s=a,b,c).

(6) $$\beta_{r}^{s}=\exp(100\;*\;\mu_{r}^{s})$$

(7) $$w_{r}^{s}=\frac{\beta_{r}^{s}}{\sum \beta_{r}^{s}}$$

(8) $$WAE(m^{s}\left(\theta \right))=\sum w_{r}^{s}m_{r}^{s}\left(\theta \right)$$

After determining the weight of each nonlinear response, many researchers [19,42] calculated the WAE according to (8) and utilized the classical Dempster combination rule to fuse multiple WAEs. However, due to the significant difference in weight distribution among the nonlinear responses in this paper, the weighted average method is no longer suitable for the fusion of two nonlinear responses. Taking the testing sample $S_{1}$ as an example, its true label is $\theta_{1}$. Under sensor a, the two nonlinear responses x and y have weights:

$$\left\{w_{x}^{a},\;w_{y}^{a}\right\}\;=\;\{0.999803,\;0.000197\}.$$

Then, the unnormalized weighted average evidence is calculated as:

$$WAE(m^{a}\left(\theta \right))\;\approx \;\{0.954,\;0.0452,\;9.16\times 10^{-4}\}.$$

Obviously, the WAE is very similar to the BPA provided by the second harmonic, which almost completely ignores all the information contained in the third harmonic, regardless of whether it is beneficial or erroneous. Furthermore, the BPA values provided by the nonlinear responses in this paper are usually extreme due to the presence of the softmax layer. Therefore, regardless of the extent to which the nonlinear response y in test sample $S_{1}$ negates label $\theta_{1}$, $m_{y}^{a}\left(\theta_{1}\right)$ → 0 is unavoidable, which makes the value of $w_{y}^{a}m_{y}^{a}\left(\theta_{1}\right)$ difficult to intervene in the fusion result effectively. In other words, the influences of extreme probability values are mitigated by the weighted average process. Therefore, we believe that using the weighted average method to preprocess the BPAs provided by them is imprudent.

(9) $$w^{s}\left(\theta \right)\left\{\begin{array}{cc} \left(1-w_{x}^{s})(1-w_{y}^{s}\right) & \mathrm{if}\;\;\;\;\;\theta \in X^{s}⋂Y^{s}\\ 1-w_{x}^{s} & \mathrm{if}\;\;\;\;\;\theta \in X^{s}\;\;\;\;\mathrm{and}\;\;\;\;\theta \notin Y^{s}\\ 1-w_{y}^{s} & \mathrm{if}\;\;\;\;\;\theta \in Y^{s}\;\;\;\;\mathrm{and}\;\;\;\;\theta \notin X^{s}\\ 1 & \mathrm{if}\;\;\;\;\;\theta \notin X^{s}⋃Y^{s} \end{array}\right.$$

(10) $${\bar{w}}^{s}\left(\theta \right)=\frac{w^{s}\left(\theta \right)}{\sum w^{s}\left(\theta \right)}$$

(11) $${\tilde{m}}^{s}\left(\theta \right)=\frac{1}{1-K^{s}}\sum_{\theta_{i}⋂\theta_{j}=\theta }{{\bar{w}}^{s}\left(\theta \right)m}_{x}^{s}\left(\theta_{i}\right)m_{y}^{s}(\theta_{j})\;$$

(12) $${\bar{m}}^{s}\left(\theta \right)=\frac{{\tilde{m}}^{s}\left(\theta \right)}{\sum {\tilde{m}}^{s}\left(\theta \right)\;}$$

To address the aforementioned issue, we transferred the weights of the nonlinear responses to all labels in $\Theta^{s}$, and a simple method for determining the label weight $w^{s}(\theta )$ was proposed as follows: First, we defined all θ that satisfy $m_{r}^{s}\left(\theta \right)<\max(m_{r}^{s}\left(\theta \right))$ to form the set R, which contains all labels with the nonhighest support from the nonlinear response r under sensor s. When r = x, R = X, and when r = y, R = Y. The higher the weight $w_{r}^{s}$ of the nonlinear response r under sensor s, the lower the weight that should be assigned to the labels it does not support. Thus, these unsupported labels should be given a weight of $1-w_{r}^{s}$. The initial weights of the labels in $\Theta^{s}$ decrease as the number of nonsupporters increases. Accordingly, all labels in $\Theta^{s}$ were divided into four categories: those supported by no nonlinear response, those supported only by nonlinear response x, those supported only by nonlinear response y, and those supported by both nonlinear responses. These categories correspond to the four conditions for calculating the initial weights as per (9). What we have to be aware of is that only the labels in the set R were penalized in terms of weight. For the labels supported by the nonlinear response, we set their initial weights to 1 by default. All labels in $\Theta^{s}$ received a final weight ${\bar{w}}^{s}\left(\theta \right)$ that sums to 1 after normalization according to (10). These final weights were added into (4) to improve the classical Dempster combination rule, resulting in (11). The calculated result ${\tilde{m}}^{s}\left(\theta \right)$ using the improved rule was normalized to obtain the BPA value ${\bar{m}}^{s}\left(\theta \right)$ provided by sensor s according to (12).

To prove the advantages of the improved rule, we take the testing sample $S_{1}$ as an example, where its true label is $\theta_{1}$. Under sensor a, the two nonlinear responses x and y have weights:

$$\left\{w_{x}^{a},\;w_{y}^{a}\right\}\;=\;\{0.999803,\;0.000197\}.$$

According to (9) and (10), the final weights of the labels are calculated as:

$$\left\{{\bar{w}}^{a}{(\theta }_{1}\right),\;{\bar{w}}^{a}{(\theta }_{2}),\;{\bar{w}}^{a}{(\theta }_{3})\}\;\approx \;\{0.999606,\;0.000197,\;0.000197\}.$$

It is easy to observe that the difference between the BPA values provided by sensor a for labels $\theta_{1}$ and $\theta_{2}$ significantly decreased. This indicates that, although the third harmonic is assigned a very low weight, its strong negation of label $\theta_{1}$ is still given consideration in the decision process rather than being ignored. There are numerous instances that can support this point. We list another testing sample $S_{2}$ to illustrate how to effectively utilize beneficial information from the third harmonic. The true label of $S_{2}$ is $\theta_{2}$, and the labels $\theta_{1}$, $\theta_{2}$, and $\theta_{3}$ satisfy:

$$\begin{array}{c} m_{x}^{a}\left(\theta \right)=\left\{m_{x}^{a}\left(\theta_{1}\right),\;m_{x}^{a}\left(\theta_{2}\right),\;m_{x}^{a}\left(\theta_{3}\right)\right\}\;=\;\{0.982,\;5.85\times 10^{-3},\;1.38\times 10^{-3}\}.\\ m_{y}^{a}\left(\theta \right)=\left\{m_{y}^{a}\left(\theta_{1}\right),\;m_{y}^{a}\left(\theta_{2}\right),\;m_{y}^{a}\left(\theta_{3}\right)\right\}\;=\;\{8.66\times 10^{-16},\;0.894,6.99\times 10^{-3}\}. \end{array}$$

The final weights of the labels are calculated as:

$$\left\{{\bar{w}}^{a}{(\theta }_{1}\right),\;{\bar{w}}^{a}{(\theta }_{2}),\;{\bar{w}}^{a}{(\theta }_{3})\}\;\approx \;\{0.999606,\;0.000197,\;0.000197\}.$$

The normalized fusion results are:

$${\bar{m}}^{a}\left(\theta \right)\;\approx \;\{8.213\times 10^{-10},\;0.9981,\;1.840\times 10^{-3}\}.$$

Obviously, the third harmonic rectifies the erroneous identification made by the second harmonic.

In summary, our improvement method proposed in this paper enhances the rationality of the decision fusion process and improves the accuracy of the decision results. Therefore, we re-fused the BPAs provided by the two nonlinear responses. The label with the highest fused BPA value was taken as the identification label. As shown in Figure 8, the mean identification accuracies over 100 training sessions for sensors a, b, and c are 93.73%, 93.13%, and 92.24%, respectively, which represent improvements of 0.51%, 0.93%, and 0.59%, respectively, compared to the classic Dempster combination rule. This proves that our improvement method proposed in this paper reasonably uses the beneficial information from different nonlinear responses, thereby enhancing the identification performance of each sensor.

### 3.5. Multi-Sensor Fusion

**Figure 10:**
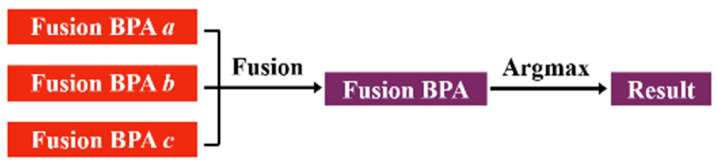
The decoupled identification framework of multi-sensor decision-level fusion for multiple subtle parameters.

The decision-level fusion of multi-harmonic nonlinear response can effectively improve the identification accuracy of each sensor. However, the relative position of each ultrasonic sensor to the micro-crack groups is fixed, resulting in the sensor receiving an ultrasonic signal with insufficient information about the micro-crack groups. In contrast, multi-sensor data fusion technologies can successfully overcome the limitations of single sensors, integrating the crack information carried by all sensors, thereby enhancing the reliability and accuracy of the identification results [43]. Based on the BPA a, BPA b, and BPA c, which are obtained by the decision-level fusion of multi-harmonic nonlinear responses under sensors a, b, and c, a sensor-level information fusion framework was established in this paper. Given that decision-level fusion performs best in the previous section, we continued to employ DS evidence theory to integrate the effective information from the three sensors, achieving the decision-level fusion of multiple ultrasonic sensors, as shown in Figure 10. Since the identification targets are still the 12 subtle parameter labels, the frame of discernment Θ is shown in (13). P(Θ) is the power set consisting of elements from Θ. From the previous part, it is easy to know that the BPA value ${\bar{m}}^{s}\left(\theta \right)$ must satisfy (14).

(13) $$\Theta =\left\{\theta_{1},\theta_{2},\theta_{3},\ldots ,\theta_{12}\right\}$$

(14) $$\left\{\begin{array}{c} {\bar{m}}^{s}\left(\Phi \right)=0\\ \sum_{\theta \subset P\left(\Theta \right)}{\bar{m}}^{s}\left(\theta \right)=1 \end{array}\right.$$

(15) $$m\left(\theta \right)=\frac{1}{1-K}\sum_{\theta_{i}⋂\theta_{j}⋂\theta_{k}=\theta }{\bar{m}}^{a}\left(\theta_{i}\right){\bar{m}}^{b}(\theta_{j}){\bar{m}}^{c}\left(\theta_{k}\right)$$

(16) $$1-K=\sum_{\theta_{i}⋂\theta_{j}⋂\theta_{k}\neq \emptyset }{\bar{m}}^{a}\left(\theta_{i}\right){\bar{m}}^{b}(\theta_{j}){\bar{m}}^{c}\left(\theta_{k}\right)$$

**Figure 11:**
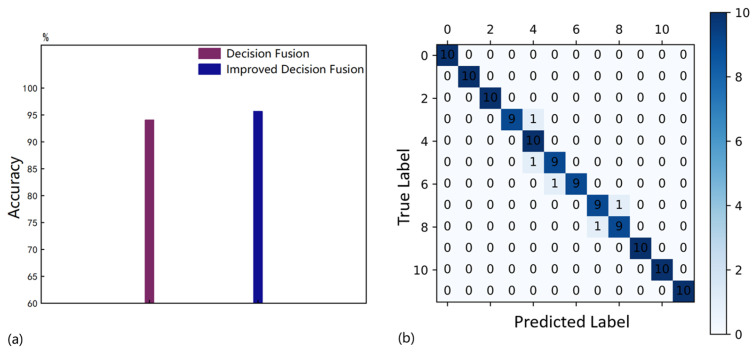
(**a**) The mean decoupled identification accuracies of quantity and size parameters. (**b**) The confusion matrix.

For the decision-level fusion of multiple ultrasonic sensors, we used the classic DS combination rule, as shown in (15), where K is the conflict coefficient between the two sensors, as shown in (16). The BPA value $m\left(\theta \right)$ for label θ is jointly determined by the three sensors. The label with the highest BPA value was taken as the identification label. It took 98.75 s to train all the 1D CNNs for 600 epochs. As shown in Figure 11a, the mean identification accuracy over 100 training sessions is 94.07%, which is only a 0.34% improvement over sensor a.

### 3.6. Improvement of Multi-Sensor Fusion

To find out the cause, we analyzed the fusion process of numerous testing samples and found that there is usually a certain degree of conflict between the BPAs provided by each sensor. The sensor with a higher conflict coefficient than others exhibits lower reliability and is more likely to provide erroneous information, which can strongly interfere with the identification results of the other sensors and increase the risks of decision fusion, even leading to counterintuitive decision results [44,45]. The classical DS evidence theory does not mitigate the negative impact caused by unreliable sensors, which greatly limits improvement in identification accuracy. Therefore, it is necessary to assign weights to all sensors based on their conflicts [46]. How to measure conflicts and calculate the sensor weights may be a difficult task. In addition to the conflict coefficient K in the classical DS evidence theory, many scholars [46,47,48,49] have proposed numerous conflict measurement methods, such as similarity degree or evidence distance. However, these methods also exhibit certain limitations when dealing with extreme probability values. Taking the testing sample $S_{3}$ as an example, whose true label is $\theta_{1}$, for sensors a, b, and c, the labels $\theta_{1}$, $\theta_{2}$, and $\theta_{3}$ satisfy:

$$\begin{array}{cc} {\bar{m}}^{a}\left(\theta \right) & =\{{\bar{m}}^{a}\left(\theta_{1}\right),\;{\bar{m}}^{a}\left(\theta_{2}\right),\;{\bar{m}}^{a}\left(\theta_{3}\right)\}\;=\;\{0.9083,\;0.0825,\;0.0042\}.\\ {\bar{m}}^{b}\left(\theta \right) & =\{{\bar{m}}^{b}\left(\theta_{1}\right),\;{\bar{m}}^{b}\left(\theta_{2}\right),\;{\bar{m}}^{b}\left(\theta_{3}\right)\}\;=\;\{1.022\times 10^{-4},\;0.9885,\;0.0056\}.\\ {\bar{m}}^{c}\left(\theta \right) & =\{{\bar{m}}^{c}\left(\theta_{1}\right),\;{\bar{m}}^{c}\left(\theta_{2}\right),\;{\bar{m}}^{c}\left(\theta_{3}\right)\}\;=\;\{3.374\times 10^{-6},\;0.0003,\;0.9992\}. \end{array}$$

It is easy to observe that the conflict between sensors a and c is significantly greater than that between sensors a and b. However, the calculated conflict coefficient between sensors a and b is $K_{ab}$ ≈ 0.9676, and between sensors a and c is $K_{ac}$ ≈ 0.9724, indicating that the conflict coefficient K does not exhibit high sensitivity to conflict. Additionally, according to the evidence distance measurement method proposed by Jousselme [48], the calculated evidence distance between sensors a and b is $d_{ab}$ ≈ 0.7614, and between sensors a and c is $d_{ac}$ = 0.7681. In fact, when ${\bar{m}}^{c}\left(\theta_{1}\right)$ → 0, ${\bar{m}}^{c}\left(\theta_{2}\right)$ → 0, and ${\bar{m}}^{c}\left(\theta_{3}\right)$ → 1, the distance $d_{ac}$ approaches a constant. Therefore, it is challenging to use Jousselme’s evidence distance to significantly represent the difference in the aforementioned conflicts.

To address the aforementioned issue, a simple method for evaluating sensor conflicts was proposed as follows: We defined θ that satisfies ${\bar{m}}^{s}\left(\theta \right)$ = $max({\bar{m}}^{s}\left(\theta \right))$ as the label most supported by sensor s, denoted as ${\hat{\theta }}_{\left(s\right)}$. For simplicity, we only considered the labels corresponding to the highest BPA values of at least one sensor. Using sensors b and c as an example: Firstly, when ${\hat{\theta }}_{\left(b\right)}$ = ${\hat{\theta }}_{\left(c\right)}$, it is evident that the conflict between sensors b and c increases as the product of ${\bar{m}}^{b}({\hat{\theta }}_{\left(c\right)})$ and ${\bar{m}}^{c}({\hat{\theta }}_{\left(b\right)})$ decreases. Simultaneously, the conflict also increases as the product of $1-{\bar{m}}^{b}({\hat{\theta }}_{\left(b\right)})$ and $1-{\bar{m}}^{c}({\hat{\theta }}_{\left(c\right)})$ decreases. Therefore, if the geometric mean of these two products decreases, we consider the conflict between sensors b and c to have increased and their weights to have decreased, which means that the weight of sensor a increases. Secondly, when ${\hat{\theta }}_{\left(a\right)}$ ≠ ${\hat{\theta }}_{\left(b\right)}$ = ${\hat{\theta }}_{\left(c\right)}$, we only consider ${\hat{\theta }}_{\left(b\right)}$. Similarly, the conflict between sensors b and c increases as the difference between ${\bar{m}}^{b}({\hat{\theta }}_{\left(b\right)})$ and ${\bar{m}}^{c}({\hat{\theta }}_{\left(c\right)})$ increases. In other words, if the ratio of $max({\bar{m}}^{b}({\hat{\theta }}_{\left(b\right)}),\;{\bar{m}}^{c}({\hat{\theta }}_{\left(c\right)}))$ to $min({\bar{m}}^{b}({\hat{\theta }}_{\left(b\right)}),\;{\bar{m}}^{c}({\hat{\theta }}_{\left(c\right)}))$ increases, we consider the conflict between sensors b and c to have increased and their weights to have decreased, which means that the weight of sensor a increases. Accordingly, we defined the conflict function f as shown in (17). $f\left(a,\;b,\;c\right)$ was used to measure the conflict between sensors b and c. Clearly, the weight of sensor a increases as the value of $f\left(a,\;b,\;c\right)$ increases. Similarly, $f\left(b,\;c,\;a\right)$ was used to measure the conflict between sensors a and c, and $f\left(c,\;a,\;b\right)$ was used to measure the conflict between sensors a and b. To prove the rationality of the conflict function f, we use the example $S_{3}$, where the calculation results are $f\left(c,\;a,\;b\right)$ ≈ 7.974 and $f\left(b,\;c,\;a\right)$ ≈ 114.9, showing that the conflict between sensors a and c is significantly greater than that between sensors a and b, which aligns better with our intuition.

(17) $$f\left(a,b,c\right)=\left\{\begin{array}{c} {\left[{\bar{m}}^{b}({\hat{\theta }}_{\left(c\right)}){\bar{m}}^{c}({\hat{\theta }}_{\left(b\right)})(1-{\bar{m}}^{b}({\hat{\theta }}_{\left(b\right)}))(1-{\bar{m}}^{c}({\hat{\theta }}_{\left(c\right)}))\right]}^{-\frac{1}{2}}\\ if\;{\hat{\theta }}_{\left(b\right)}\neq {\hat{\theta }}_{\left(c\right)}\\ {\left[\frac{1-\max\left({\bar{m}}^{b}({\hat{\theta }}_{\left(b\right)}),{\bar{m}}^{c}({\hat{\theta }}_{\left(c\right)})\right)}{1-\min\left({\bar{m}}^{b}({\hat{\theta }}_{\left(b\right)}),{\bar{m}}^{c}({\hat{\theta }}_{\left(c\right)})\right)}\right]}^{-1}\\ if\;{\hat{\theta }}_{\left(a\right)}\neq {\hat{\theta }}_{\left(b\right)}={\hat{\theta }}_{\left(c\right)}\\ 1\\ if\;{\hat{\theta }}_{\left(a\right)}={\hat{\theta }}_{\left(b\right)}={\hat{\theta }}_{\left(c\right)} \end{array}\right.\;$$

(18) $$SUM=f\left(a,b,c\right)+f\left(b,c,a\right)+f\left(c,a,b\right)\;$$

(19) $$F\left(a,b,c\right)=\frac{f\left(a,b,c\right)}{SUM}$$

(20) $$W_{a}=F\left(a,b,c\right)$$

(21) $$W_{b}=F\left(b,c,a\right)$$

(22) $$W_{c}=F\left(c,a,b\right)$$

(23) $$WAE(m\left(\theta \right))=\sum W_{s}{\bar{m}}^{s}\left(\theta \right)$$

(24) $$m\left(\theta \right)={\left(WAE(m\left(\theta \right))⊕\;WAE(m\left(\theta \right))\ldots WAE(m\left(\theta \right))\right)}_{k}$$

The purpose of measuring conflicts is to determine the weights of the sensors, as shown in (18)–(22). We propose a method of normalizing the conflict functions to assign the sensor weights $W_{a}$, $W_{b}$, and $W_{c}$. Although each sensor’s weight may vary greatly, this variation largely stems from the extreme probability values in the BPAs provided by sensors. Unlike the previous part, there are only two nonlinear responses in the decision fusion process. Discussing the concept of conflict in the context of information fusion with only two evidence bodies is meaningless. However, for multi-sensor decision fusion, the presence of extreme probability values usually causes a significant conflict between a sensor and the others, thereby reducing its weight. Based on the comprehensive analysis, we believe that weighting the BPA provided by each sensor to obtain the WAE is feasible. Finally, according to (23) and (24), by applying the classical Dempster combination rule, the WAEs were merged for k times (k = 5), which was applied to the decoupled identification of subtle parameters of micro-crack groups. As shown in Figure 11a, the mean identification accuracy over 100 training sessions is 95.68%, which is 1.61% higher than before the improvement. This proves that our proposed improvement method in this paper mitigates the negative impact of conflicts to some extent and enhances the identification performance of the multi-sensor decision fusion method. Figure 11b shows a confusion matrix of any one identification result with an identification accuracy of 95.8%.

The simulation results presented in this paper preliminarily demonstrate the excellent performance of the proposed multi-harmonic and multi-sensor fusion methods. Undoubtedly, environmental noise, as well as the nonlinearities in material and equipment, may lead to suboptimal ultrasonic signals, which could, to some extent, affect the initial identification accuracy prior to fusion. However, we can reasonably speculate on the contribution of these methods in actual detection. On the one hand, regardless of the quality of the actual ultrasonic signals or the initial BPA, the sensitivity-weighted decision fusion method can effectively integrate multi-harmonic BPAs. On the other hand, the limited number of actual samples can lead to extreme BPA; however, the conflict-weighted multi-sensor fusion method can still enhance the rationality of the decisions. Therefore, this paper theoretically presents a feasible fusion-based recognition method, and future work will focus on validating the effectiveness of these approaches in practical detection.

## 4. Conclusions

This paper proposed a novel method for identifying the quantity and size parameters using nonlinear ultrasonics coupled with convolutional neural networks (CNNs). Our methods enhance identification accuracy through a two-level fusion of nonlinear responses and sensor data. The key findings and contributions of this research are as follows: (1) The one-dimensional convolutional neural network (1D CNN) achieves remarkably high accuracy in identifying the quantity parameter. (2) For decoupled identification of quantity and size parameters, compared to a direct fusion of nonlinear responses at the data, feature, and decision levels, our improved weighted Dempster–Shafer (DS) evidence theory effectively enhances identification accuracy. (3) Compared to the existing conflict measurement methods, our new conflict measurement method significantly highlights the difference between pieces of evidence with extreme probability values, leading to a more rational fusion of highly conflicting sensors and further enhancing identification accuracy. While these results represent a significant advancement in the field of micro-crack group characterization, we acknowledge certain limitations and areas for future research: (1) The information fusion methods we proposed in theory still require future experimental validation. (2) We did not consider the impacts of the changes in ultrasonic excitation frequency and amplitude on identification accuracy. We did not consider the influence of different orientation angles and positions of micro-cracks. Future work should investigate these effects to enhance the robustness of our proposed method across diverse testing conditions. (3) Our proposed conflict measurement method is presently limited to scenarios involving three sensors. To extend the applicability of this approach, future research should focus on generalizing the conflict function to accommodate a variable number of sensors.

## Figures and Tables

**Table 1:** The quantity and size parameter labels.

The Quantity Parameter Labels		The Size Parameter Labels	
A	[10, 100]	D	[0.22 mm, 0.40 mm]
B	[110, 200]	E	[0.42 mm, 0.60 mm]
C	[210, 300]	F	[0.62 mm, 0.80 mm]
-	-	G	[0.82 mm, 1.00 mm]

**Table 2:** The average ANP of the second harmonic.

	D	E	F	G
A	0.032	0.045	0.061	0.071
B	0.034	0.047	0.058	0.059
C	0.035	0.048	0.067	0.072

**Table 3:** The average ANP of the third harmonic.

	D	E	F	G
A	0.016	0.017	0.018	0.019
B	0.014	0.023	0.028	0.028
C	0.017	0.018	0.022	0.022

## Data Availability

Data are contained within the article.
